# Ubiquitin-dependent proteolysis in yeast cells expressing neurotoxic proteins

**DOI:** 10.3389/fnmol.2015.00008

**Published:** 2015-03-12

**Authors:** Ralf J. Braun

**Affiliations:** Institut für Zellbiologie, Universität BayreuthBayreuth, Germany

**Keywords:** ubiquitylation, ubiquitin-proteasome system, autophagy, ubiquitin-dependent vesicular trafficking, neurodegeneration, cell death, *Saccharomyces cerevisiae*

## Abstract

Critically impaired protein degradation is discussed to contribute to neurodegenerative disorders, including Parkinson's, Huntington's, Alzheimer's, and motor neuron diseases. Misfolded, aggregated, or surplus proteins are efficiently degraded via distinct protein degradation pathways, including the ubiquitin-proteasome system, autophagy, and vesicular trafficking. These pathways are regulated by covalent modification of target proteins with the small protein ubiquitin and are evolutionary highly conserved from humans to yeast. The yeast *Saccharomyces cerevisiae* is an established model for deciphering mechanisms of protein degradation, and for the elucidation of pathways underlying programmed cell death. The expression of human neurotoxic proteins triggers cell death in yeast, with neurotoxic protein-specific differences. Therefore, yeast cell death models are suitable for analyzing the role of protein degradation pathways in modulating cell death upon expression of disease-causing proteins. This review summarizes which protein degradation pathways are affected in these yeast models, and how they are involved in the execution of cell death. I will discuss to which extent this mimics the situation in other neurotoxic models, and how this may contribute to a better understanding of human disorders.

## Introduction

Ubiquitin is a highly conserved protein with 76 amino acids (Weissman et al., 2011; Finley et al., 2012). It is covalently linked to lysine side chains of substrate proteins by the sequential action of the ubiquitin-activating enzyme (E1), ubiquitin-conjugating enzymes (E2), and substrate-specific ubiquitin ligases (E3) (Weissman et al., 2011; Finley et al., 2012). Deubiquitylating enzymes (DUBs) recycle ubiquitin, replenishing the cellular pool of free ubiquitin. The high variety of ubiquitin modifications, including mono- and polyubiquitylations, destines the degradation, localization, and/or function of substrate proteins. Consequently, numerous cellular processes are regulated by ubiquitylation, including protein degradation, cell death control, and vesicular trafficking (Weissman et al., 2011; Finley et al., 2012). These ubiquitin-dependent processes are highly conserved from yeast to humans (Finley et al., 2012).

Polyubiquitylated proteins are degraded within the proteasome, a cylindrical multiprotein complex with chymotrypsin-, trypsin-, and caspase-like proteolytic activities. This ubiquitin-dependent degradation of proteins via the proteasome is called the ubiquitin-proteasome system (UPS) (Weissman et al., 2011; Finley et al., 2012). The ubiquitylation of many plasma membrane proteins promotes their targeting into endosomes and multivesicular bodies (MVB) leading to their degradation by multiple proteases in the lysosomes (or vacuoles in yeast) (Finley et al., 2012; MacGurn et al., 2012). This MVB pathway of protein degradation (also called endosomal-lysosomal pathway) is a ubiquitin-controlled vesicle-based protein degradation pathway, which is independent from proteasomes. Ubiquitylation can also be involved in the degradation of substrate proteins via autophagy (Kuang et al., 2013; Lu et al., 2014). Autophagy is a cellular process where proteins, protein aggregates, or organelles are enclosed by a double membrane forming autophagosomes, which eventually fuse with lysosomes (vacuoles) for degradation. One mechanism to ensure specificity during autophagy relies on the ubiquitylation of target proteins or organelles and the consequent use of specific adaptors that connect the ubiquitin system with the autophagy pathway (Kuang et al., 2013; Lu et al., 2014). The UPS, the MVB pathway, and autophagy share some components, such as the AAA-ATPase p97/VCP (or Cdc48 in yeast) (Bug and Meyer, 2012; Dargemont and Ossareh-Nazari, 2012), and the E3 ligase Nedd4 (or Rsp5 in yeast) (MacGurn et al., 2012; Fang et al., 2014; Lu et al., 2014), making these proteins to potential key players that decide by which pathway a protein is to be degraded.

Accumulation of aggregated proteins is a common hallmark of many neurodegenerative disorders, and believed to contribute to neuronal dysfunction (Lansbury and Lashuel, 2006). In Parkinson's disease (PD) cytoplasmic Lewy bodies are protein aggregates mainly comprised by the protein α-synuclein (Uversky, 2007), and nuclear protein aggregates of the polyglutamine protein hungtingtin are typical for Huntington's disease (HD) (Ross and Tabrizi, 2011). In Alzheimer's disease (AD) the hydrophobic peptide β-amyloid is produced in cells but accumulates in extracellular plaques (Laferla et al., 2007). The microtubule-associated protein tau (MAPT), and UBB^+1^, the frameshift variant of human ubiquitin B, is enriched in intracellular inclusions during AD (van Leeuwen et al., 1998; Mandelkow and Mandelkow, 2012). Accumulation of cytoplasmic aggregates of disease-causing proteins, such as TDP-43 or FUS/TLS, occurs during the motor neuron disease amyotrophic lateral sclerosis (ALS) (Andersen and Al-Chalabi, 2011). Most of these disease-associated proteins or aggregates are ubiquitylated. Therefore, the observed accumulation of protein aggregates has been explained by dysfunctional protein degradation pathways, including the UPS and autophagy (Dennissen et al., 2012; Dantuma and Bott, 2014). However, the precise role of ubiquitin-dependent proteolysis and its importance for the progression of the human disorders remains poorly understood.

Yeast is an established model for measuring cytotoxicity and programmed cell death, and for dissecting conserved mechanisms of apoptosis and necrosis (Carmona-Gutierrez et al., 2010). Diverse roles of ubiquitin-dependent protein degradation have been described in distinct yeast cell death scenarios. The ubiquitin-dependent and proteasome-independent routing of misfolded proteins to the MVB pathway for their degradation protects cells from cytotoxicity (Wang et al., 2011). Elevated proteasome capacity extends the replicative life span and fitness of yeast cells, which are more resistant against proteotoxic stress (Kruegel et al., 2011). Decreased proteasome capacity by proteasome inhibition leads to disturbances in the amino acid homeostasis, thereby executing cell death (Suraweera et al., 2012). Impairment of distinct branches of the UPS pathway, including the ER- and the mitochondrion-associated degradation (ERAD/MAD) are sufficient to trigger cell death in yeast, emphasizing their cytoprotective role in the homeostasis of the ER and mitochondria (Braun et al., 2006; Zischka et al., 2006; Heo et al., 2010). However, UPS impairment (due to proteasome inhibition) can also prevent from cell death, *e.g*., when cell death is triggered by acetic acid and the chemotherapeutic drug cisplatin, respectively (Valenti et al., 2008; Cunha et al., 2013). Thus, ubiquitin-dependent proteolysis is involved in both the execution of and the prevention from yeast cell death.

In recent years, many yeast models have been established to analyze the influence of human neurotoxic protein expression on yeast cell survival, including models for PD, HD, AD, and ALS (Gitler, 2008; Miller-Fleming et al., 2008; Winderickx et al., 2008; Bharadwaj et al., 2010; Braun et al., 2010; Khurana and Lindquist, 2010; Bastow et al., 2011; Mason and Giorgini, 2011). Here, I summarize how ubiquitin-dependent protein degradation is impaired in yeast cell death models expressing neurotoxic proteins, and which role proteolysis plays in the execution of cell death. Further, I will discuss some similarities between the yeast models expressing neurotoxic proteins, and the animal and cell culture disease models.

## Yeast models expressing neurotoxic proteins

### Parkinson's disease (PD)

PD is the most prevalent age-related movement disorder characterized by a progressive loss of dopaminergic neurons in the substantia nigra, leading to the impairment of normal motor function culminating in resting tremor, bradykinesia and rigidity (Lees et al., 2009). In most familiar and sporadic cases, PD is associated with Lewy bodies, *i.e*., intracellular cytoplasmic aggregates composed of the protein α-synuclein (Uversky, 2007). Missense mutations in the *SNCA* gene, resulting in the expression of α-synuclein variants (A18T, A29S, A30P, A53T, E46K, H50Q, G51D), as well as duplication and triplication of *SNCA*, leading to elevated α-synuclein levels, are causative for PD in some familiar forms of the disorder (Fujioka et al., 2014). Numerous α-synuclein disease models expressing wild-type and disease-associated variants have been established, including several yeast models.

#### In yeast, α-synuclein is a membrane-associated protein, which is degraded via the UPS, autophagy, and potentially the MVB pathway

When expressed in yeast, α-synuclein binds to vesicles of the secretory pathway, leading to its localization to the plasma membrane (Outeiro and Lindquist, 2003; Dixon et al., 2005; Sharma et al., 2006; Zabrocki et al., 2008). This depends on α-synuclein phosphorylation (Basso et al., 2013; Tenreiro et al., 2014), and can be interrupted by genetic manipulation (*e.g*., α-synuclein-A30P) (Outeiro and Lindquist, 2003; Dixon et al., 2005; Sharma et al., 2006). Upon high expression levels α-synuclein forms cellular aggregates in a nucleation-dependent manner, which starts at the plasma membrane and eventually leads to cytoplasmic inclusions (Outeiro and Lindquist, 2003). These inclusions co-localize with markers of different vesicles, including Ypt1 (ER-to-Golgi), Ypt31 (late Golgi), Sec4 (secretory vesicles-to-plasma membrane), Ypt6 (endosome-to-Golgi), Vps21 and Ypt52 (early-to-late endosome) and Ypt7 (late endosome-to-vacuole) (Gitler et al., 2008). Thus, α-synuclein is an aggregation-prone membrane-associated protein.

Although α-synuclein is ubiquitylated in yeast (Outeiro and Lindquist, 2003), to which extent the UPS contributes to its degradation in human cells (Xilouri et al., 2013) or more specifically in yeast (see below), remains an open debate. Treatment of yeast cells expressing α-synuclein with the proteasome inhibitor lactacystin resulted in increased α-synuclein aggregation (Zabrocki et al., 2005; Lee et al., 2008). Consistently, expression of α-synuclein in the yeast strain *sen3-1*, which harbors a mutation in the gene encoding the regulatory proteasome subunit Rpn2, led to increased steady-state levels of α-synuclein (using untagged α-synuclein) and to increased formation of aggregates (using GFP-tagged α-synuclein) (Sharma et al., 2006). These data suggest that α-synuclein is a UPS substrate in yeast.

In other studies with yeast cells expressing GFP-tagged α-synuclein, α-synuclein aggregate clearance was neither affected by treatment with the proteasome inhibitor MG132 (Petroi et al., 2012; Tenreiro et al., 2014), nor by mutation in the gene encoding the regulatory proteasome subunit Rpt6 (*cim3-1*) (Petroi et al., 2012). Further, the steady-state levels of α-synuclein were not affected by MG132 treatment (Tenreiro et al., 2014). These data argue against the contribution of the UPS in α-synuclein degradation in yeast. Here, α-synuclein aggregate clearance was dependent on autophagy and vacuolar protease activity. Treatment of yeast cells expressing α-synuclein-GFP with the protease inhibitor phenylmethylsulfonyl fluoride (PMSF), an inhibitor of vacuolar proteases, resulted in a significant reduction of this clearance (Petroi et al., 2012). Similarly, genetic interruption of autophagy (Δ*atg1* or Δ*atg7*) delayed aggregate clearance (Petroi et al., 2012; Tenreiro et al., 2014), and increased the steady-state levels of α-synuclein (Δ*atg7*) (Tenreiro et al., 2014). Consistently, inducing autophagy by rapamycin promoted aggregate removal (Zabrocki et al., 2005), confirming that α-synuclein aggregates are degraded via autophagy.

Aggregate clearance was not limited to autophagy, because aggregate clearance still took place in the absence of autophagy and upon very low UPS activity (*cim3-1* Δ*atg1* strain), suggesting for an additional cellular clearing mechanism independent from autophagy and the UPS (Petroi et al., 2012). Indeed, the yeast E3 ligase Rsp5, and its homolog Nedd4 in mammalian cells, play critical roles in α-synuclein degradation (Tofaris et al., 2011). In yeast, α-synuclein was identified as an Rsp5 target for ubiquitylation, and upon *RSP5* mutation (*rsp5-1* strain), both the steady-state level of α-synuclein, as well as the number of cells showing α-synuclein aggregation were increased as compared to wild-type strain (Tofaris et al., 2011). Although Rsp5/Nedd4 is critically involved in many cellular processes, including the MVB pathway, ubiquitin-dependent autophagy and the proteasome-dependent degradation of misfolded proteins (MacGurn et al., 2012; Fang et al., 2014; Lu et al., 2014), there are some line of evidence suggesting that the MVB pathway contributes to α-synuclein degradation (Tofaris et al., 2011). Mammalian Nedd4 promotes the degradation of endogenous α-synuclein by lysosomes, and the targeting of α-synuclein to the lysosomes depends on the endosomal sorting complex (ESCRT) (Tofaris et al., 2011). Due to the high conservation of protein degradation pathways between mammalian cells and yeast, the critical contribution of Rsp5 in α-synuclein degradation in yeast might suggest for a potential role of the MVB pathway in α-synuclein degradation, besides the UPS and autophagy.

#### α-synuclein expression in yeast leads to impairment of the UPS, and vesicular trafficking

Expression of α-synuclein in yeast leads to UPS impairment. The protein composition of the proteasome is altered, concomitant to a moderate decrease in the chymotrypsin-like enzymatic proteasomal activity (in isolated proteasomes), and to a marked delay in the degradation of short-lived proteins (pulse-chase assay) (Chen et al., 2005). Consequently, the cellular levels of polyubiquitylated proteins increased moderately (Chen et al., 2005). The degradation of the UPS model substrate GFPu, in which the C-terminus of this protein comprises a degron, was delayed upon α-synuclein expression in yeast (Outeiro and Lindquist, 2003). The impairment of UPS-dependent protein degradation upon α-synuclein expression appears to be substrate specific. Whereas, the degradation of the cytosolic proteasome substrate Deg1-β-Gal was unaffected, the degradation of the ER luminal substrate CPY^*^ but not of the ER membrane substrate sec61-2 was severely impaired (Cooper et al., 2006). Thus, α-synuclein expression impairs the UPS and more specifically the degradation of selective substrates of the ER-associated degradation (ERAD) pathway.

α-Synuclein expression also affects vesicular trafficking, including ER to Golgi transport, endocytosis, vesicular recycling back to the plasma membrane, and vacuolar fusion (Cooper et al., 2006; Gitler et al., 2008; Zabrocki et al., 2008; Basso et al., 2013). Since the MVB pathway and autophagy depend both on vesicular fusion processes, it is very likely that α-synuclein expression also affects the protein degradation via these two vesicle-based pathways. Measuring the degradation rates of substrates of autophagy or the MVB pathway upon α-synuclein expression will help to address this issue.

#### α-synuclein expression in yeast triggers cell death, which is modulated by the activities of the UPS, autophagy, and ubiquitin-dependent vesicular trafficking

Yeast cells overexpressing wild-type and disease-associated α-synuclein demonstrated growth deficits and age-dependent loss of clonogenic cell survival paralleled by the emergence of morphological markers of apoptosis and necrosis (Willingham et al., 2003; Flower et al., 2005; Witt and Flower, 2006; Büttner et al., 2008, 2013a,b; Lee et al., 2008; Su et al., 2010). The cellular accumulation of ROS and mitochondrial dysfunction are pivotal for the execution of α-synuclein-triggered cell death. The use of the antioxidant *N*-acetyl cysteine (NAC) or the use of yeast strains deleted for mitochondrial DNA (ρ^0^ strain) protected from ROS and α-synuclein-triggered cell death (Büttner et al., 2008, 2013a). The translocation of the mitochondrial cell death proteins Nuc1 and cytochrome *c*, into the nucleus and the cytosol, respectively, was observed to be critical for the execution of cell death (Flower et al., 2005; Büttner et al., 2013b). The ER also contributes to cytotoxicity, because α-synuclein expression results in ER stress and in the induction of the unfolded protein response (UPR) (Cooper et al., 2006).

Since α-synuclein has been proposed to be a UPS substrate and since α-synuclein expression resulted in UPS impairment (especially ERAD), it is likely that the UPS is involved in modulating α-synuclein-triggered cytotoxicity. Moderate α-synuclein expression, which is non-toxic for wild-type yeast cells, resulted in severe growth deficits in yeast cells bearing mutations in the 20S proteasomal barrel (*pre1-1001, pre2-1001, doa3-1*) (Dixon et al., 2005; Sharma et al., 2006) and in the 19S regulatory particle of the 26S proteasome (*sen3-1*) (Sharma et al., 2006), or treated with the proteasome inhibitor lactacystin (Lee et al., 2008). Consistently, expression of Rpt5, a component of the 19S regulatory particle, and expression of the ERAD ubiquitin ligase Hrd1 suppressed α-synuclein-triggered cytotoxicity (Liang et al., 2008; Gitler et al., 2009). Bridging high-throughput genetic and transcriptional data with the ResponseNet algorithm predicted the AAA-ATPase Cdc48, also critically involved in ERAD, to be a modulator of α-synuclein-triggered cytotoxicity (Yeger-Lotem et al., 2009). Thus, the UPS in general, and specifically the ERAD pathway appears to be a potent modulator of α-synuclein-triggered cell death.

Since α-synuclein aggregates have been proposed to be substrates of autophagy, it is likely that autophagy, like the UPS, modulates α-synuclein-triggered cytotoxicity. In fact, the *in silico* combination of high-throughput genetic and transcriptional data predicted the target of rapamycin (TOR) pathway, as a modulator of α-synuclein-triggered cytotoxicity (Yeger-Lotem et al., 2009). Addition of the TOR-inhibitor rapamycin markedly enhanced the growth deficits elicited by α-synuclein (Yeger-Lotem et al., 2009). Since inactivation of the TOR pathway induces autophagy, these data suggested, that enhancing autophagy is harmful but not cytoprotective for cultures expressing α-synuclein. Consistently, pharmacological inhibition of autophagy by treatment with chloroquine markedly extended chronological life span of yeast cells expressing α-synuclein (Sampaio-Marques et al., 2012). Although rapamycin also affects other cellular pathways, it remains possible that autophagy, in contrast to the UPS, plays a detrimental role in α-synuclein-triggered cytotoxicity.

Besides the UPS and autophagy, ubiquitin-dependent vesicle trafficking plays a role in modulating α-synuclein-triggered cytotoxicity. The E3 ligase Rsp5, involved in ubiquitin-dependent vesicle trafficking, was predicted by the ResponseNet algorithm to affect α-synuclein-triggered cytotoxicity (Yeger-Lotem et al., 2009). Indeed, loss-of-function mutations in the gene encoding Rsp5 (*rsp5-1* strain) increased α-synuclein-triggered growth deficits, whereas overexpression of Rsp5 was cytoprotective (Tofaris et al., 2011). Chemical genetic screens in wild-type yeast cells established that *N*-aryl benzimidazole (NAB) promoted endosomal transport and protected cells from cytotoxicity (Tardiff et al., 2013). This was dependent on the deubiquitinase Doa4, the E3 ubiquitin ligase Rsp5, the Rsp5 adaptor Bul1, the DUBs Ubp7, and Ubp11, which can deubiquitylate Rsp5 substrates, the potential Rsp5 substrates (Bap2, Bap3, and Mmp1), and Vps23, which directs Rsp5 substrates for degradation in the vacuole (Tardiff et al., 2013). Notably, promoting ER-Golgi vesicle trafficking had very similar effects: α-synuclein-triggered cytotoxicity was reduced, and this reduction also depended on ubiquitin proteases, namely Ubp3 and its co-factor Bre5 (Cooper et al., 2006; Gitler et al., 2008). Thus, promoting ubiquitin-regulated vesicle trafficking prevents α-synuclein-triggered cytotoxicity.

#### Yeast α-synuclein models are highly useful to elucidate the role of diverse ubiquitin-related protein degradation pathways in modulating cytotoxicity and neuronal cell death

**Figure 1:**
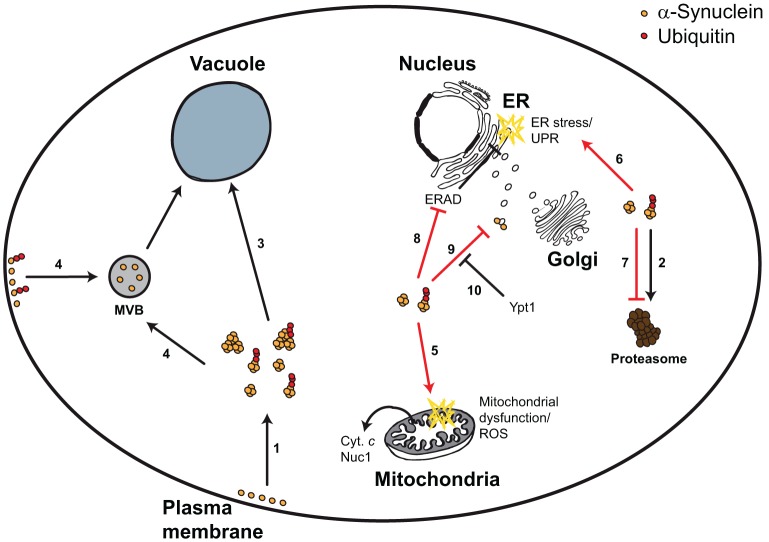
**Yeast model for α-synuclein-triggered cytotoxicity**. α-Synuclein is a plasma membrane- and vesicle-bound protein that upon high expression levels or upon mutation forms smaller and larger aggregates, which can be ubiquitylated (1). α-Synuclein can be degraded via the UPS (2), autophagy (3), and potentially via the MVB pathway (4). Aggregated α-synuclein triggers mitochondrial dysfunction, ROS, and mitochondrion-dependent cell death (5), as well as ER stress and the UPR (6). These cytotoxic effects can at least partially be explained by α-synuclein-dependent inhibition of the proteasome (7), the ERAD pathway (8), or vesicular trafficking (9). Impaired vesicular trafficking includes (but is not limited to) ER-to-Golgi transport, which can be efficiently restored by Ypt1 expression (10).

In yeast, α-synuclein is both a substrate and an inhibitor for the UPS, autophagy, and the ubiquitin-dependent vesicular trafficking (Figure 1, Table 1). The activities of the UPS and of MVB pathways appear to play protective roles, whereas increased autophagy potentially contribute to α-synuclein-triggered cytotoxicity. The relevance of the different pathways in modulating α-synuclein-triggered cytotoxicity might depend on α-synuclein itself, *e.g*., the expression levels, the post-translational modifications, the cellular localizations, folding or distinct aggregation conditions. Similarly, the chronological and replicative aging of the yeast cultures might be decisive. Systematic analyses of these factors will help to get a better understanding of the pathophysiological effects of α-synuclein expression in yeast with respect to the reciprocal effects to ubiquitin-dependent protein degradation.

**Table 1:** **Ubiquitin-dependent protein degradation in yeast models expressing neurotoxic proteins**.

**Yeast model**	**Neurotoxic protein**	**Hallmarks of neurotoxic protein**	**Degradation of neurotoxic protein**	**Impairment of protein degradation**	**Cytotoxicity of neurotoxic protein**	**Modulation of cytotoxicity by protein degradation pathways**	**Selected references**
PD	α-Synuclein	Membrane and vesicle-associated; Aggregation upon high expression levels in the cytoplasm	Substrate of the UPS, autophagy, and the MVB pathway	*UPS impairment:* Increased levels of polyubiquitylated proteins; delay in the degradation of short-lived proteins and UPS model substrates; specific inhibition of the ERAD pathway *Autophagy and MVB impairment:* n.d.	Growth deficit; Loss of clonogenic survival; Emergence of ROS; Mitochondrial dysfunction and mitochondrion-dependent cell death; ER stress and UPR induction	Promoting UPS and ERAD relieve cytotoxicity; Putative detrimental role of autophagy; Promoting vesicle trafficking including the MVB pathway relieves cytotoxicity	Outeiro and Lindquist, 2003; Chen et al., 2005; Dixon et al., 2005; Flower et al., 2005; Zabrocki et al., 2005; Cooper et al., 2006; Sharma et al., 2006; Büttner et al., 2008; Lee et al., 2008; Liang et al., 2008; Zabrocki et al., 2008; Gitler et al., 2009; Yeger-Lotem et al., 2009; Su et al., 2010; Tofaris et al., 2011; Petroi et al., 2012; Sampaio-Marques et al., 2012; Basso et al., 2013; Büttner et al., 2013a,b; Tardiff et al., 2013; Tenreiro et al., 2014
HD	Huntingtin exon 1 polyQ (with and without proline-rich domain)	Cytoplasmic, aggregation-prone protein; Very tight aggregates predominate in the case of 103QP; Amorphous aggregates predominate in the case of 103Q; Aggregates directed into aggresomes or perivacuolar inclusions (IPOD)	Ubiquitylated polyQ as substrates for the UPS, and for ubiquitin-dependent autophagy; Non-ubiquitylated IPOD-localized polyQ as autophagy substrate? Role of endocytosis or MVB pathway in polyQ degradation?	*UPS impairment:* Increased levels of polyubiquitylated proteins;specific inhibition of the UFD, ERAD, APC pathways (103Q);impairment of Sis1-dependent protein degradation (96QP) *Autophagy impairment:* n.d. *MVB pathway:* impairment of endocytosis	Growth deficit; Emergence of ROS; Mitochondrial dysfunction; ER stress and UPR induction	Promoting ERAD relieves cytotoxicity; Impaired endocytosis increases cytotoxicity; Promoting Sis1-dependent protein degradation relieves cytotoxicity; Impairment of ubiquitin-dependent autophagy increases cytotoxicity	Meriin et al., 2002, 2003; Duennwald et al., 2006a,b; Sokolov et al., 2006; Solans et al., 2006; Meriin et al., 2007; Bocharova et al., 2008; Duennwald and Lindquist, 2008; Giorgini et al., 2008; Kaganovich et al., 2008; Bocharova et al., 2009; Wang et al., 2009; Ocampo et al., 2010; Kruegel et al., 2011; Tauber et al., 2011; Park et al., 2013; Lu et al., 2014
AD	β-Amyloid	β-Amyloid (or GFP-Aβ) directed into the cytosol	n.d.	Impairs mitochondrial pre-protein maturation	Moderate growth deficit (for GFP-Aβ); Moderate ROS production, loss of mitochondrial membrane potential, and decreased oxygen consumption	n.d.	Caine et al., 2007; Mossmann et al., 2014
		β-Amyloid directed into the secretory pathway	Clioquinol promotes Aβ degradation	Impairs clathrin-mediated endocytosis	Growth deficit	*PICALM* and clioquinol as suppressors of cytotoxicity	Treusch et al., 2011; D'Angelo et al., 2013; Matlack et al., 2014
	Tau	Hyperphosphorylated cytoplasmic aggregates	n.d.	n.d.	Increases α-synuclein-induced growth deficit	n.d.	Vandebroek et al., 2005; Zabrocki et al., 2005; Vandebroek et al., 2006; Vanhelmont et al., 2010; De Vos et al., 2011
	UBB^+1^	Ubiquitylated and truncated	Substrate of the UPS	*UPS impairment:* Increased levels of polyubiquitylated proteins; delay in the degradation of UFD and N-end rule substrates; impairment of DUB activities	Increases polyQ-induced growth deficit; Apoptosis, necrosis, emergence of ROS, and loss of clonogenic survival upon prolonged expression	Promoting mitochondrion-associated degradation (MAD) prevents mitochondrial impairment and cell death	Tank and True, 2009; Verhoef et al., 2009; Dennissen et al., 2011; Krutauz et al., 2014; Braun et al., 2015
ALS	TDP-43	Cytoplasmic aggregation-prone protein	n.d.	n.d.	Growth deficit; Loss of clonogenic survival; Emergence of ROS; Pivotal mitochondrial dysfunction	Cdc48^TS^ as enhancer of TDP-43-triggered cytotoxicity; Promoting vesicular trafficking partially rescues cytotoxicity	Johnson et al., 2008, 2009; Braun et al., 2011; Armakola et al., 2012; Tardiff et al., 2013

*n.d.: not determined*.

These studies are very promising, due to the high consistence of the yeast α-synuclein models with other α-synuclein model systems. In mammalian cells, α-synuclein degradation also depends on the UPS, autophagy, and ubiquitin-dependent vesicular trafficking (more specifically the MVB pathway) (Tofaris et al., 2011; Ebrahimi-Fakhari et al., 2012; Xilouri et al., 2013). Likewise, in mammalian cells, α-synuclein interferes with these ubiquitin-modulated protein degradation pathways, and the activities of these pathways are discussed to influence the cytotoxicity of α-synuclein (Ebrahimi-Fakhari et al., 2012; Xilouri et al., 2013). Yeast α-synuclein models have already been very valuable in deciphering novel cellular mechanisms linking ubiquitin-dependent pathways with α-synuclein-triggered cytotoxicity. For instance, the influence of the ubiquitin-modulated ER-Golgi vesicle trafficking on α-synuclein-triggered cytotoxicity was first identified in yeast and then confirmed in flies and worms (Cooper et al., 2006). Likewise, the degradation of α-synuclein via the MVB pathway is conserved from yeast to mammalians (Tofaris et al., 2011), and the protective effect of promoting ubiquitin-dependent endosomal trafficking by NAB was first identified in yeast and later on confirmed in worms, rats, and human PD patient-derived neurons (Chung et al., 2013; Tardiff et al., 2013).

### Huntington's disease (HD)

HD is an autosomal dominant neurodegenerative disorder characterized by a progressive loss of neurons in the striatum and the cortex with a consequent decline of cognitive and motor functions (Ross and Tabrizi, 2011). HD is caused by an abnormal polyglutamine (polyQ) expansion in the protein huntingtin due to an aberrant CAG codon expansion in the exon 1 of the gene encoding huntingtin (Ross and Tabrizi, 2011). This results in an aggregation-prone protein eventually triggering cytotoxicity and neuronal cell loss (Ross and Tabrizi, 2011). Increasing the length of the polyQ expansion accelerates aggregation of huntingtin and strictly correlates with the increase in cytotoxicity and the decrease in disease onset (Ross and Tabrizi, 2011). In order to dissect underlying mechanisms, various HD models have been established, comprising transgenic mouse lines, mammalian cell culture, and yeast (Mason and Giorgini, 2011; Ross and Tabrizi, 2011).

#### In yeast, huntingtin with disease-associated expanded glutamine stretches (polyQ) is a cytoplasmic aggregation-prone protein, which is degraded via the UPS and autophagy

In yeast, expression of fluorescence protein-tagged huntingtin exon 1 with disease-inducing polyQ expansions (*e.g*., 103Q) led to very efficient cytoplasmic aggregation, in contrast to fusion proteins with normal glutamine repeats (*e.g*., 25Q) (Meriin et al., 2002, 2003; Duennwald et al., 2006a,b). Intermediate polyQ length (*e.g*., 47Q) showed moderate aggregation when expressed in logarithmically growing cells but here strong aggregation occurred delayed upon chronological aging (Cohen et al., 2012). Besides the length of polyQ expansions, aggregation was influenced by the amino acid sequences flanking the polyQ stretch (Duennwald et al., 2006b; Wang et al., 2009), and by the presence of endogenous proteins with prion properties and glutamine repeats (Duennwald et al., 2006a). The N-terminal domain of huntingtin (N17 fragment) which precedes the polyQ stretch is required for recruiting the chaperonin TRiC and the 14-3-3 protein Bmh1 which both promote aggregation in yeast and cell-free systems (Tam et al., 2009; Wang et al., 2009; Duennwald, 2011; Crick et al., 2013). PolyQ followed by the endogenous proline-rich region of huntingtin (103QP) tended to form very tight aggregates (one or two per cell), whereas polyQ lacking the proline-rich region (103Q) preferred to form amorphous aggregates dispersed throughout the cytosol (Duennwald et al., 2006b). The yeast protein Rnq1 co-localized with both 103QP and 103Q aggregates, and its prion conformation [RNQ^+^] was found to be necessary for aggregation of 103QP and 103Q (Duennwald et al., 2006a).

In line with the appearance of different types of polyQ aggregates, these aggregates are directed to at least two cellular compartments. First, misfolded polyQ (103QP) was actively transported to aggresomes (Wang et al., 2009), which are large juxtanuclear aggregates that co-localize with the centrosomes in mammalian cells and the spindle pole bodies in yeast (Johnston et al., 1998; Wang et al., 2009). In mammalian cells, aggresomes are ubiquitylated, highly dynamic and easily accessible to the UPS (Johnston et al., 1998). They are formed from smaller aggregates, which are actively transported to the centrosome via the microtubule cytoskeleton (Johnston et al., 1998). Both the N17 fragment and the proline-rich domain of huntingtin are required for aggresome formation in yeast (Wang et al., 2009). Alternatively, polyQ aggregates (it is unclear whether 103Q or 103QP has been used) could be transported to perivacuolar inclusions, called “insoluble protein deposits” (IPOD) (Kaganovich et al., 2008). IPODs are non-ubiquitylated insoluble protein aggregates covered by the ubiquitin-like autophagy protein Atg8 (Kaganovich et al., 2008). Therefore, it is tempting to speculate that polyQ aggregates in perivacuolar inclusions are substrates of autophagy, whereas polyQ aggregates in aggresomes are primarily substrates of the UPS.

PolyQ aggregates could be ubiquitylated (96QP) (Lu et al., 2014), and physically interacted with proteins of the UPS, including proteasomal subunits (103QP, 96QP) (Wang et al., 2009; Park et al., 2013). Consistently, polyQ aggregate clearance (103Q) was improved in a yeast strain with elevated UPS capacities (Δ*ubr2*), confirming that polyQ aggregates are substrates of the UPS (Kruegel et al., 2011). However, the degradation of ubiquitylated polyQ was not limited to the UPS but could also occur by ubiquitin-dependent autophagy (Lu et al., 2014). Here, polyQ (96QP) was ubiquitylated by the E3 ligase Rsp5, and ubiquitylated polyQ was recognized by the ubiquitin-Atg8 adaptor protein Cue5, enabling the targeting of polyQ aggregates (96QP) to the vacuole for their degradation (Lu et al., 2014). In sum, ubiquitylated polyQ aggregates are degraded via the UPS, and ubiquitin-dependent autophagy.

Yeast strains with mutations affecting the formation of endocytic vesicles demonstrated decreased aggregation of polyQ (47Q, 103Q, 103QP) (Meriin et al., 2007). In contrast, lack of proteins which are pivotal for later steps of endocytosis increased polyQ aggregation (103Q), and when present these proteins co-localized with polyQ aggregates (103Q) (Meriin et al., 2007). These data suggest that these ubiquitin-controlled vesicular processes are involved in the formation of polyQ aggregates (Meriin et al., 2007). However, whether they are also involved in the degradation of polyQ remains elusive.

#### PolyQ expression leads to impairment of specific branches of the UPS pathway in yeast

PolyQ expression inhibits the UPS. In the presence of polyQ, increased cellular levels of polyubiquitylated proteins were observed (with 103Q but not with 103QP) (Duennwald and Lindquist, 2008), genes involved in ubiquitin cycle and protein ubiquitylation were up-regulated (with 103Q) (Giorgini et al., 2008; Tauber et al., 2011), and the degradation of cytosolic and ER-associated proteins were impaired (with 103Q but not with 103QP) (Duennwald and Lindquist, 2008). Notably, the polyQ-dependent UPS impairment is substrate specific. Upon polyQ expression (103Q), the degradation of cytosolic ubiquitin-fusion degradation (UFD) substrates (Ub-P-LacZ) was more affected than the degradation of cytosolic substrates of the N-end rule pathway (Ub-R-LacZ) (Duennwald and Lindquist, 2008). Consistently, the degradation of ER-associated proteins via ERAD, which shares many components with the UFD pathway, was drastically affected upon polyQ expression (103Q) (Duennwald and Lindquist, 2008). Here, the degradation of the ER luminal misfolded variant of the carboxypeptidase Y (CPY^*^) was impaired, as well as the degradation of the misfolded ER membrane protein sec61-2, and others (Duennwald and Lindquist, 2008). PolyQ (both 103Q and 103QP) physically interacted with the AAA-ATPase Cdc48 and its co-factors Ufd1 and Npl4, which are pivotally involved in UFD and ERAD (Duennwald and Lindquist, 2008; Wang et al., 2009). It has been proposed that sequestration of the Cdc48-Ufd1/Npl4 complex by polyQ (103Q) is the cause for the specific impairment of the UFD and ERAD pathways, culminating in ER stress and UPR induction (Duennwald and Lindquist, 2008). Notably, the interaction of polyQ (103QP) with the Cdc48-Ufd1/Npl4 complex was essential for the formation of the polyQ-containing aggresomes (Wang et al., 2009). The formation of these aggresomes is believed to be a protective cellular mechanism to prevent the evenly spread of misfolded proteins within a cell (Johnston et al., 1998). Therefore, the interaction of the Cdc48-Ufd1/Npl4 complex with polyQ might be both protective (at least for 103QP) and detrimental (for 103Q); it prevents from the accumulation of misfolded polyQ but promotes specific dysfunction of the UFD and ERAD pathways.

Expression of polyQ containing the proline-rich domain (97QP) can also lead to UPS dysfunction via an alternative mechanism, which is based on the sequestration of chaperones. PolyQ (97QP) inhibited the UPS-mediated degradation of the misfolded cytosolic variant of carboxypeptidase Y (ΔssCPY^*^) lacking the signaling sequence (ss) for entering the secretory pathway (Park et al., 2013). Upon polyQ expression (97QP), the steady-state levels and the turnover rates of ΔssCPY^*^ were increased and delayed, respectively (Park et al., 2013). PolyQ-triggered (97QP) UPS inhibition was not due to direct interference with proteasomal function, because the ubiquitin-independent proteasomal degradation of ornithine decarboxylase was not affected by polyQ expression (Park et al., 2013). UPS dysfunction could also not be explained by interference of polyQ (97QP) with the ubiquitylation of ΔssCPY^*^ prior its proteasomal degradation (Park et al., 2013). Instead, polyQ (97QP) interfered with the transfer of the misfolded protein to the proteasome, prior proteasomal degradation, which surprisingly took place in the nucleus (Park et al., 2013). The nuclear transfer of misfolded ΔssCPY^*^ was done by binding to the type II Hsp40 chaperone Sis1, which shuttles into the nucleus, and polyQ (97QP) interfered with this process by sequestering Sis1 in the cytosol (Park et al., 2013). Consistently, Sis1 overexpression restored the degradation of ΔssCPY^*^ in the presence of polyQ. Thus, polyQ (both 103Q and 97QP) interferes with the proteasome-dependent degradation of proteins in the nucleus and the cytosol, by sequestering the Sis1 chaperone (for 97QP) and the Cdc48-Ufd1/Npl4 complex (for 103Q), respectively.

Whether polyQ interferes with autophagy and protein degradation based on ubiquitin-controlled vesicular transport remains to be determined. Since polyQ aggregates (103Q) impaired endocytosis in yeast (Meriin et al., 2003, 2007), it is tempting to speculate that polyQ aggregates (103Q) also affect these vesicle-based protein degradation pathways.

#### PolyQ expression in yeast triggers mitochondrial dysfunction and ER stress, which is modulated by specific UPS activities

PolyQ constructs encoding 103 glutamine residues (103Q) efficiently triggered growth deficits and morphological markers of apoptosis in yeast (Meriin et al., 2002; Duennwald et al., 2006a; Sokolov et al., 2006; Solans et al., 2006). In contrast, polyQ constructs encoding 25 glutamine residues (25Q) remained non-toxic, and the constructs with intermediate polyQ lengths showed intermediate cytotoxicity (Meriin et al., 2002; Duennwald et al., 2006a; Sokolov et al., 2006; Solans et al., 2006; Ocampo et al., 2010). Besides the number of glutamine expansions, the cytotoxicity of polyQ strongly depended on the proline-rich domain flanking the polyQ stretch (Duennwald et al., 2006b). PolyQ (103QP) remained non-toxic, whereas polyQ lacking the proline-rich region (103Q) induced cytotoxicity (growth deficits) (Duennwald et al., 2006b). In this context, the presence of the prion conformation of the yeast protein Rnq1 was a prerequisite for polyQ-triggered cytotoxicity (103Q), and the presence of other endogenous yeast proteins with glutamine repeats also influenced polyQ-triggered cytotoxicity (103Q) (Duennwald et al., 2006a). Thus, polyQ-triggered cytotoxicity depends on the length of the glutamine repeats, the absence or presence of the proline-rich domain, and the cellular protein interaction network.

Cytotoxic polyQ (103Q) physically interacted with mitochondria and triggers critical mitochondrial dysfunction (Solans et al., 2006; Ocampo et al., 2010). PolyQ expression (103Q but not 103QP) also induced ER stress leading to UPR (Duennwald and Lindquist, 2008), and has also been proposed to lead to lethal impairment of cell cycle progression (Bocharova et al., 2008, 2009). ER and cell cycle impairments are believed to be consequences of the impairment of specific branches of the UPS pathway by polyQ expression (103Q), including the ERAD and anaphase promoting complex (APC) pathways (Bocharova et al., 2008, 2009; Duennwald and Lindquist, 2008). The mitochondrion-associated protein degradation pathway (MAD) shares pivotal components with the ERAD pathway (Heo et al., 2010; Taylor and Rutter, 2011). Therefore, it is tempting to speculate that polyQ expression (103Q) also impairs MAD, contributing to the observed mitochondrial damage.

PolyQ-triggered cytotoxicity was increased by genetic impairment of the ERAD and UPR pathways and by application of ER stress (for 103Q but not for 103QP) (Duennwald and Lindquist, 2008). In contrast, promoting ERAD by expression of Npl4 and Ufd1, which are pivotally involved in ERAD, or constitutive activation of the UPR suppressed polyQ-triggered cytotoxicity (103Q) (Duennwald and Lindquist, 2008). These data are in line with the idea that ERAD dysfunction by polyQ expression (103Q) is critical in the execution of cytotoxicity and cell death. Deletion of the APC substrate *ASE1* relieved polyQ-triggered cytotoxicity (103Q), suggesting that preventing the accumulation of Ase1 upon dysfunction of the APC pathway is beneficial (Bocharova et al., 2008). Expression of polyQ flanked by the proline-rich domain (96QP), which is non-toxic under normal conditions, became cytotoxic upon accumulation of the UPS model substrate ΔssCPY^*^ and could be relieved upon Sis1 overexpression (Park et al., 2013). Genetic inactivation of ubiquitin-dependent autophagy (*e.g*., *rsp5-2*, Δ*cue5*) also induced cytotoxicity upon polyQ expression (96QP) (Lu et al., 2014). These data suggest that besides ERAD and APC pathways, the Sis1-dependent protein degradation and the ubiquitin-dependent autophagy are pivotal in the modulation of polyQ-triggered cytotoxicity in yeast.

#### Yeast polyQ models are highly useful to elucidate the role of diverse ubiquitin-related protein degradation pathways in modulating cytotoxicity and neuronal cell death

**Figure 2:**
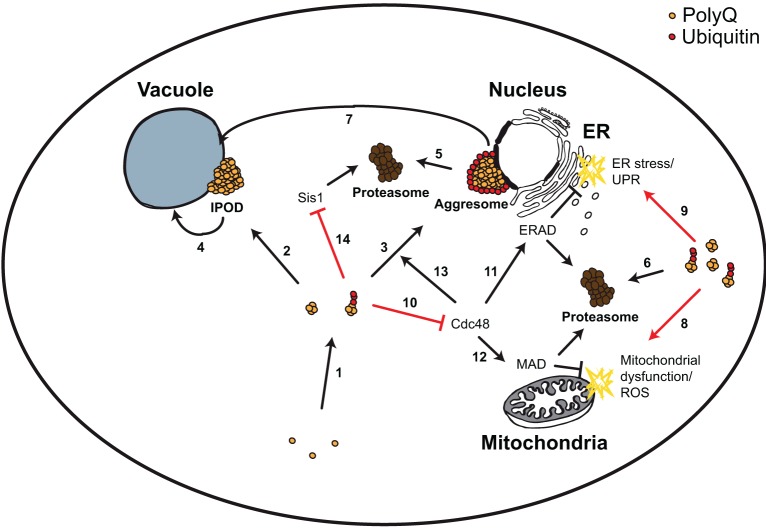
**Yeast model for polyQ-triggered cytotoxicity**. PolyQ monomers are very aggregation-prone forming smaller and larger aggregates, which can be ubiquitylated (1). These aggregates can be transported to two cellular compartments, the perivacuolar inclusion (IPOD, non-ubiquitylated) (2) or the perinuclear aggresome (ubiquitylated) (3). IPODs are potential substrates of autophagy (4). Aggresomes and other ubiquitylated polyQ aggregates are substrates of the UPS (5, 6), and of ubiquitin-dependent autophagy (via the Cue5 ubiquitin-Atg8 adaptor protein) (7). PolyQ aggregates trigger mitochondrial dysfunction and ROS (8), as well as ER stress and UPR (9). The first was explained by a direct detrimental physical interaction of polyQ aggregates with mitochondria. For the latter it has been shown that polyQ aggregates sequester the AAA-ATPase Cdc48 (10), leading to ERAD dysfunction (11). Sequestering Cdc48 could potentially lead to MAD dysfunction (12) (which shares many components with ERAD) and to impairment of aggresome formation (13) (which depends on Cdc48). Ubiquitylated polyQ aggregates inhibit the degradation of misfolded cytosolic proteins in the nucleus via sequestering the chaperone Sis1 (14).

PolyQ expression in yeast leads to aggregation, and aggregates can be allocated to distinct cellular compartments, including aggresomes and perivacuolar inclusions (IPOD) (Figure 2, Table 1). Aggregated polyQ is a substrate of the UPS and autophagy. Besides its role of a substrate of protein degradation, polyQ impairs the UPS (especially ERAD), and also affects proper function of vesicular transport (more specifically endocytosis). This leads to ER stress, mitochondrial dysfunction, and cell death. All these features depend on the length of the polyQ stretch, the absence (or presence) of the N-terminal part (N17 fragment) and the proline-rich domain, respectively, and the cellular protein interaction network.

The data obtained in yeast HD models have been very helpful for a better understanding of polyQ-triggered effects in higher model systems, as well as in humans. For instance, the allocation of different aggregates into distinct cellular compartments (aggresomes and IPOD, respectively) was conserved from yeast to mammalians (Johnston et al., 1998; Kaganovich et al., 2008; Wang et al., 2009). As in yeast, polyQ are substrates of the UPS and autophagy (Lu et al., 2014; Margulis and Finkbeiner, 2014; Martin et al., 2014). PolyQ aggregates block specific branches of the UPS (including the ERAD and the Sis1-dependent pathways) in mammalians as in yeast (Duennwald and Lindquist, 2008; Park et al., 2013; Margulis and Finkbeiner, 2014). Like in yeast cells, polyQ impairs vesicle-based protein degradation, including autophagy (Sapp et al., 1997; Aronin et al., 1999; Meriin et al., 2003, 2007; Martin et al., 2014).

Interestingly, in cultured cells and in flies, ubiquitylation (and sumolyation) of the N-terminal part of huntingtin (N17) at specific residues (K6, K9, and K15) turned out to be an efficient modulator of polyQ aggregation and neurotoxicity (Steffan et al., 2004). Thus, distinct (poly)ubiquitylation patterns could on the one hand dictate the fate of polyQ (*e.g*., aggregation and degradation) and on the other hand determine its effects on ubiquitin-dependent degradation of other cellular proteins. This issue could further be dissected in yeast HD models.

Of specific interest is the role of p97/VCP, the ortholog of the yeast AAA-ATPase Cdc48. As in yeast, p97/VCP and its cofactors Npl4 and Ufd1 were sequestered by polyQ aggregates in mammalian cells leading to ERAD dysfunction, ER stress, and UPR induction (Duennwald and Lindquist, 2008). Overexpression of Npl4, Ufd1, or p97/VCP rescued from ERAD dysfunction and/or ER stress and UPR (Duennwald and Lindquist, 2008; Leitman et al., 2013). In *C. elegans*, p97/VCP was involved in the clearance of detrimental polyQ aggregates (Nishikori et al., 2008). Since p97/VCP has also been proposed to play important roles in autophagy and apoptosis (Braun and Zischka, 2008; Krick et al., 2010), it is tempting to speculate that the interaction of polyQ aggregates with this protein complex is critical for the switch from UPS and autophagy dysfunctions to neuronal cell loss.

### Alzheimer's disease (AD)

AD is the most prevalent form of age-related dementia (Querfurth and Laferla, 2010). Synaptic loss and neuronal decline can be observed in affected brain regions, including the hippocampus and the cortex (Querfurth and Laferla, 2010). The accumulation of extracellular senile plaques and intracellular aggregates, composed of β-Amyloid, and the intracellular accumulation of neurofibrillary tangles comprising hyperphosphorylated variants of the protein tau and UBB^+1^, the frameshift variant of ubiquitin B, contribute to AD progression (van Leeuwen et al., 1998; Laferla et al., 2007; Benilova et al., 2012; Mandelkow and Mandelkow, 2012).

#### Yeast β-amyloid models

Several yeast models expressing β-Amyloid in the cytosol and in the secretory pathway have been generated (Caine et al., 2007; Treusch et al., 2011; D'Angelo et al., 2013; Matlack et al., 2014; Mossmann et al., 2014) (Table 1). Cytosolic β-Amyloid (GFP-Aβ42 or Aβ42-GFP fusion proteins) led to moderate growth deficits and to the induction of the heat shock response in wild-type cells (Caine et al., 2007). Mitochondria isolated from aging-prone yeast cells (Δ*coa6*) upon prolonged exposure to cytosolic Aβ42 demonstrated signs of mitochondrial impairment, including inhibition of mitochondrial pre-protein maturation, increased ROS production, decreased mitochondrial membrane potential and reduced oxygen consumption (Mossmann et al., 2014). Recent studies suggest that mitochondrial impairment triggers UPS dysfunction and *vice versa* (Livnat-Levanon et al., 2014; Maharjan et al., 2014; Segref et al., 2014; Braun et al., 2015). Since mitochondrial and UPS dysfunction are believed to contribute to AD progression, it is of high interest, whether cytosolic Aβ42 interacts with ubiquitin-dependent processes.

Whereas, the detrimental effects of cytosolic β-Amyloid remained moderate, β-Amyloid directed to the secretory pathway resulted in high cytotoxicity concomitant to β-Amyloid oligomerization and aggregation (Treusch et al., 2011; D'Angelo et al., 2013; Matlack et al., 2014). Here, β-Amyloid impaired the clathrin-mediated endocytic trafficking of a plasma membrane receptor (which is a ubiquitin-modulated process), and expression of endocytic genes rescued β-Amyloid-triggered cytotoxicity (Treusch et al., 2011; D'Angelo et al., 2013). A genome-wide screen for modifiers of β-Amyloid-triggered cytotoxicity identified the yeast homolog of phosphatidylinositol binding clathrin assembly protein (*PICALM*), as suppressor of cytotoxicity (Treusch et al., 2011). A screen of ~140,000 compounds for β-Amyloid rescue in yeast identified the 8-hydroxyquinoline clioquinol as effective in preventing β-Amyloid-triggered growth deficits via promoting β-Amyloid degradation and restoring endocytic function (Matlack et al., 2014). The outcome of these two unbiased yeast screens validate the usefulness of yeast AD models, because *PICALM* is one of the most highly validated AD risk factors (Treusch et al., 2011), and clioquinol is effective in both mouse and *C. elegans* AD models (Matlack et al., 2014).

#### Yeast tau models

Yeast models expressing AD-associated tau have been generated (De Vos et al., 2011) (Table 1). Tau forms oligomers and is hyperphosphorylated when expressed in yeast as it is in AD patients (Vandebroek et al., 2005, 2006; Zabrocki et al., 2005; Vanhelmont et al., 2010). Although tau expression *per se* remained non-toxic in wild-type cells, it increased α-synuclein-triggered growth deficits (Zabrocki et al., 2005). Therefore, combining several AD risk factors (*e.g*., tau, β-Amyloid, and/or UBB^+1^) could lead to more effective yeast AD models. This turned out to be necessary in mouse AD models, because mutations in tau are also not very toxic in transgenic mice (Nisbet et al., 2015). Similarly, AD-associated mutations in the β-Amyloid precursor protein (APP) or the γ-secretase components presenilin 1 or 2, which are pivotally involved in β-Amyloid generation from APP, do also not fully recapitulate the key pathological events of AD (Nisbet et al., 2015). In order to mimic simultaneously more than one pathological aspect of AD double and triple transgenic AD mouse models or AD patient-derived neuronal stem-cell-derived three-dimensional culture systems have been developed (van Tijn et al., 2012; Choi et al., 2014; Nisbet et al., 2015).

#### Yeast UBB^+1^ models

UBB^+1^-expressing yeast models have been established (Tank and True, 2009; Verhoef et al., 2009; Dennissen et al., 2011; Krutauz et al., 2014; Braun et al., 2015) (Table 1). As in mammalian cells, UBB^+1^ is a substrate of the UPS in yeast. It is ubiquitylated prior proteasomal degradation and truncated by the DUB Yuh1 (or mammalian ubiquitin carboxy-terminal hydrolase UCH-L3) (De Vrij et al., 2001; van Tijn et al., 2007, 2010; Tank and True, 2009; Verhoef et al., 2009; Dennissen et al., 2011; Braun et al., 2015). Accumulation of UBB^+1^ impairs the UPS in yeast and mammalian cells, culminating in the accumulation of polyubiquitylated proteins. In both cases, the degradation of UFD substrates (*e.g*., Ub-G76V-GFP, Ub-P-LacZ), and of N-end rule substrates (*e.g*., Ub-R-GFP, Ub-R-LacZ) was significantly reduced (Lindsten et al., 2002; van Tijn et al., 2007; Tank and True, 2009; Braun et al., 2015). The inhibitory effect of UBB^+1^ on the UPS is at least partially due to inhibition of DUBs, especially Ubp6/USP14, which is associated with the 26S proteasome and involved in the disassembly of polyubiquitin tags from substrate proteins (Krutauz et al., 2014).

Despite of the UBB^+1^-triggered UPS impairment, both yeast and mammalian cells can tolerate UBB^+1^ without marked signs of cytotoxicity (Hope et al., 2003; van Tijn et al., 2007, 2012; Tank and True, 2009; Yim et al., 2014), and in some cases UBB^+1^ expression was even protective when mammalian cells were treated with chemicals inducing oxidative stress (Hope et al., 2003; Yim et al., 2014). Consistently, transgenic expression of UBB^+1^ in mice failed to cause overt neurodegeneration although it did affect spatial reference memory and caused a central dysfunction of respiratory regulation (Fischer et al., 2009; van Tijn et al., 2011; Irmler et al., 2012). In contrast, prolonged expression of high levels of UBB^+1^ induced cell death and pivotal mitochondrial impairment in neuronal cells and in yeast (De Vrij et al., 2001; Tan et al., 2007; Braun et al., 2015). In yeast the UBB^+1^-triggered cell death could be prevented by specifically promoting the UPS activity at mitochondria (mitochondrion-associated degradation, MAD), which protected cells from mitochondrial impairment but did not alter the steady-state levels of UBB^+1^ (Braun et al., 2015). The accumulation of UBB^+1^ increased the cytotoxicity of polyQ (103Q) in yeast (Tank and True, 2009), which is highly comparable to mammalian systems, where UBB^+1^ expression accelerates cell death in transgenic HD mouse models (de Pril et al., 2004, 2007, 2010). Thus, on the one hand UBB^+1^ is a neurotoxic protein by itself, whose cytotoxicity depends both on the cellular UPS capacity and on mitochondrial (dys)function. On the other hand UBB^+1^ is putatively a potent modifier of cytotoxicity of other misfolded neurotoxic proteins. Especially when combined with further AD risk factors, including β-Amyloid and tau, the yeast UBB^+1^ model could be very valuable for elucidating the molecular connections among AD risk factors, UPS (dys)function, mitochondrial activity, and cell survival.

### Amyotrophic lateral sclerosis (ALS)

ALS is a frequent degenerative motor neuron disease, resulting in muscle weakness and wasting (Andersen and Al-Chalabi, 2011). Among the most common ALS-associated genes are *TARDBP*, and *FUS*, encoding the RNA-binding proteins TDP-43 and FUS/TLS (Andersen and Al-Chalabi, 2011). ALS-associated variants of these proteins demonstrate mislocalization and/or a high tendency for aggregation, and these aggregates are ubiquitylated (Andersen and Al-Chalabi, 2011; Da Cruz and Cleveland, 2011). Yeast models expressing ALS-associated wild-type and mutant TDP-43 and FUS/TLS have been established to further analyze the detrimental roles of these proteins on cell survival (Bastow et al., 2011).

TDP-43 and FUS/TLS efficiently form cytoplasmic aggregates when expressed in yeast cells triggering cytotoxicity and cell death (Johnson et al., 2008; Braun et al., 2011; Kryndushkin et al., 2011; Sun et al., 2011) (Table 1). It is very little known via which pathways these neurotoxic proteins and their aggregates are degraded in yeast, whether these proteins impair ubiquitin-modulated protein degradation, and whether the activities of ubiquitin-modulated proteolysis pivotally affect their cytotoxicity. In the case of TDP-43, a temperature-sensitive variant of the AAA-ATPase Cdc48 was found as an enhancer of TDP-43-triggered cytotoxicity (growth deficits) (Armakola et al., 2012). Promoting vesicular trafficking (ER -> Golgi) by treating TDP-43-expressing yeast cells with NAB relieved TDP-43-triggered cytotoxicity (Tardiff et al., 2012, 2013). Thus, the activities of ubiquitin-modulated vesicular transport and Cdc48-dependent cellular processes may be pivotal in modulating TDP-43-triggered cytotoxicity in yeast and neurons. Cdc48 and its mammalian homolog p97/VCP are involved in many different ubiquitin-modulated processes, including ERAD, MAD, and vesicular transport, such as endocytosis, and autophagy (Bug and Meyer, 2012). Indeed, mutations in p97/VCP lead to cytoplasmic TDP-43 aggregation and cell death in neurons of transgenic mice and flies (Gitcho et al., 2009; Johnson et al., 2010; Ritson et al., 2010; Rodriguez-Ortiz et al., 2013). Yeast TDP-43 models could help to further discriminate the role of the distinct Cdc48/p97/VCP pathways in modulating cell death and neuronal loss.

## Conclusions and outlook

In yeast models expressing neurotoxic proteins, these proteins are on the one hand substrates of distinct protein degradation pathways, and on the other hand the trigger for their impairment. Consistently, the activities of the UPS, autophagy, and other ubiquitin-controlled vesicle-based protein degradation pathways are pivotal for the cytotoxicity of neurotoxic proteins. Best described for yeast PD and HD models, these findings could be confirmed in other neurotoxic model systems, confirming that yeast models expressing neurotoxic proteins are very helpful in elucidating novel paradigms of pathobiology in neurodegenerative disorders. It is very likely, that yeast models for AD (β-Amyloid, tau, UBB^+1^), and ALS (*e.g*., TDP-43, FUS/TLS), will lead to the identification of further ubiquitin-regulated protein degradation pathways that potentially underlie neuronal dysfunction and cell loss. In this respect, the easy and fast combination of different neurotoxic proteins in one yeast model (*e.g*., different AD-associated proteins) could be a very straightforward approach.

The role of free ubiquitin homeostasis has been largely unattended when analyzing the effects of ubiquitylated neurotoxic proteins and ubiquitin-dependent protein degradation on neuronal survival. For instance, loss of free ubiquitin was found in schizophrenia (Rubio et al., 2013), and down-regulation of free ubiquitin was determined to be causative for p53 accumulation and apoptosis in hippocampal neurons from rats (Tan et al., 2000). In contrast, free ubiquitin levels increase in cancer cells, which turned out to be pivotal for cell growth (Oh et al., 2013). Consistently, high levels of free ubiquitin confers resistance to inhibitors of protein translation in yeast (Hanna et al., 2003). Modulating free ubiquitin levels in yeast models expressing human neurotoxic proteins could address the role of free ubiquitin homeostasis on the degradation and the cytotoxicity of these proteins.

### Conflict of interest statement

The author declares that the research was conducted in the absence of any commercial or financial relationships that could be construed as a potential conflict of interest.

## Abbreviations

AD: Alzheimer's disease
ALS: Amyotrophic lateral sclerosis
DUB: Deubiquitylating enzyme
ERAD: ER-associated degradation
HD: Huntington's disease
IPOD: Insoluble protein deposit/ perivacuolar aggregates
MAD: Mitochondrion-associated degradation
MVB: Multivesicular bodies
PD: Parkinson's disease
polyQ: Proteins with abnormal glutamine expansions
ROS: Reactive oxygen species
UFD: Ubiquitin-fusion degradation pathway
UPR: Unfolded protein response
UPS: Ubiquitin-proteasome system.
